# Mothers’ cafeteria diet induced sex-specific changes in fat content, metabolic profiles, and inflammation outcomes in rat offspring

**DOI:** 10.1038/s41598-021-97487-x

**Published:** 2021-09-17

**Authors:** Julia Matuszewska, Tomasz Zalewski, Adam Klimaszyk, Kamil Ziarniak, Stefan Jurga, Agata Chmurzynska, Joanna H. Sliwowska

**Affiliations:** 1 Laboratory of Neurobiology, Faculty of Veterinary Medicine and Animal Science, Poznan University of Life Sciences, Wojska Polskiego 71C, 60-625 Poznan, Poland; 2 NanoBioMedical Centre, Adam Mickiewicz University, Wszechnicy Piastowskiej 3, 61-614 Poznan, Poland; 3 Department of Macromolecular Physics, Faculty of Physics, Adam Mickiewicz University, Uniwersytetu Poznańskiego 2, 61-614 Poznan, Poland; 4 Institute of Human Nutrition and Dietetics, Poznań University of Life Sciences, Wojska Polskiego 31, 60-624 Poznan, Poland

**Keywords:** Developmental biology, Physiology, Diseases, Endocrinology, Physics

## Abstract

“Western diet” containing high concentrations of sugar and fat consumed during pregnancy contributes to development of obesity and diabetes type 2 in offspring. To mimic effects of this diet in animals, a cafeteria (CAF) diet is used. We hypothesized that CAF diet given to rats before, and during pregnancy and lactation differently influences fat content, metabolic and inflammation profiles in offspring. Females were exposed to CAF or control diets before pregnancy, during pregnancy and lactation. At postnatal day 25 (PND 25), body composition, fat contents were measured, and blood was collected for assessment of metabolic and inflammation profiles. We have found that CAF diet lead to sex-specific alterations in offspring. At PND25, CAF offspring had: (1) higher percentage of fat content, and were lighter; (2) sex-specific differences in levels of glucose; (3) higher levels of interleukin 6 (IL-6), interleukin-10 (IL-10) and tumor necrosis factor (TNF-α); (4) sex-specific differences in concentration of IL-6 and TNF-α, with an increase in CAF females; (5) higher level of IL-10 in both sexes, with a more pronounced increase in females. We concluded that maternal CAF diet affects fat content, metabolic profiles, and inflammation parameters in offspring. Above effects are sex-specific, with female offspring being more susceptible to the diet.

## Introduction

Obesity results from energy imbalance, which is often associated with an increased calorie intake and reduction in physical activity. Macronutrient composition of diets contribute to development of obesity^1^. So-called “Western diet” consumed mostly in highly developed countries is an example of unhealthy eating habits. It is characterized by high intakes of butter, fried foods, high-fat dairy products, eggs, refined grains, potatoes and high-sugar drinks^2^. To imitate effects of such a diet in laboratory settings, the cafeteria (CAF) diet is used. It consists of a variety of products, which are characterized by good taste with high energy density with about 45–55% of the energy coming from fats^3–5^. Among the commonly used ingredients in this diet there are: biscuits, potato chips, peanut butter, chocolate, chocolate bars, cheese, sausage, jam, muffins, cakes, dried fruit^3,5,6^. CAF diet very well mimics “fast-food meals” popular among people^7^. It leads to development of obesity, characterized by increased body weight, changes in metabolic profiles, and inflammation^3–5^.

Influence of an “unhealthy diet” can start already in utero, and according to the prenatal programming theory, environmental factor such as diet of mother influences fetus during its prenatal development, and reprogramming of the neuroendocrine system. As a result, it may lead to the development of obesity, diabetes type 2 (DM2) and cardiovascular diseases. A strong correlation was found between low birth weight, high cortisol levels, later development of hypertension and DM2^8,9^. The offspring of mothers who suffer from diabetes during pregnancy are at higher risk of developing obesity and abnormal glucose metabolism not only in childhood, but also during adolescence and adulthood^10^. Exposure to unhealthy diets can lead to remodeling and changes in many offspring’s organs. However, these effects are dependent on the composition of the obesogenic diet, its duration, timing of exposure, and sex of offspring^11,12^.

In the light of above evidence, effective methods of assessment and control of body weight and composition are invaluable. Non-invasive techniques, such as magnetic resonance imaging (MRI)^13–18^ and nuclear magnetic resonance (NMR) are used^19–21^ to study these changes. Above methods allow to examine abnormal fat contents, fat thickness, as well as body composition, and help in prediction of development of metabolic disorders, which may appear later in life^22^.

Similarly to people consuming a “Western type of diet”, in adult animals CAF diet promotes compulsive food intake and rapid weight gain, increases fat content and induction of obesity^5,23–25^. Mice kept for 15 weeks on a CAF diet had higher body weight and abdominal fat content when compared not only to control, but also to animals fed high-fat (HF) diet^25^. CAF diet also results in alterations of metabolic profile, induces lipogenesis and decreases very-low-density lipoprotein (VLDL) export leading to lipid accumulation in the liver and hepatic steatosis^26–29^. These changes are accompanied by increased concentrations of cholesterol, glucose, and triacylglycerol in blood^24,30–32^. Finally, CAF diet leads to abdominal and visceral fat accumulation and insulin resistance^24,27,30^. In animal models, similar to situation observed in obese individuals, CAF diet-induced obesity results in inflammation^25,33,34^.

Exposure to a CAF diet during pregnancy and/or lactation also influences body weight and metabolic profile of offspring. Administration of CAF diet to mothers for 10–15 weeks altered body weight, elevated glucose, insulin, leptin and triglyceride levels^35–37^. Moreover, sex specific differences in levels of glucose and insulin in offspring after exposure of dams to a CAF diet were found^35–37^ Yet, results of studies are not consistent and depends on many variables such as duration of the diet, its content, time of exposure, species and strain of animals.

Moreover, pups from mothers consuming a CAF diet during lactation had lower body weight and lean mass, but greater fat accumulation, compared to controls at 3 months of age. Thus, it was proposed that feeding mothers a CAF diet leads to a thin-outside-fat-inside phenotype in the offspring^37^. Similarly, at weaning (PND 21) rat offspring from mothers kept on a CAF diet before pregnancy, during pregnancy and lactation had lower body weight, but higher percentage of body fat content compared to control^38^. Additionally, both male and female CAF offspring fed for 6 weeks after weaning control diet remained lighter than control, however, there was no longer any difference in percentage of fat mass between groups^38^. However, in another experimental paradigm, when rats were given a CAF diet for a longer time—before pregnancy, during pregnancy and lactation, and post-weaning to offspring, there was no increase in body weight at puberty (PND 30), but animals had higher weigh at adulthood (PND 120) and no difference in visceral adipose tissue weight was reported in male rats^6^. Thus, differences in discussed parameters depend on duration of diet exposure as well as sex of offspring.

Maternal obesity is also associated with a state of chronic, low-grade inflammation, characterized by elevated adipose tissue and systemic proinflammatory cytokine levels, and adipose tissue macrophage accumulation^39^. These changes extend to the placenta, which suggests that maternal obesity exposes the fetus to an inflammatory environment during its development^40–43^. Indeed, numerous investigators reported changes in cytokine levels in the maternal and fetal/placental compartments (for review see^39^).

CAF diet also leads to inflammatory process in adult rodents. Fifteen weeks administration of a CAF diet elevated serum level of interleukin 6 (IL-6) in male mice^25^, and administration of Western diet for 18 weeks to male rats increases level of tumor necrosis factor α (TNF-α) and decreased levels of interleukin 10 (IL-10)^44^. On the other hand, shorter exposure (for 6 weeks) to a CAF diet had no influence on inflammatory parameters (such as concentration of TNF-α and IL-6) in male rats^34^.

Effects of prenatal exposure to a CAF diet on inflammatory processes were also studied^45,46^. CAF diet given to females before and during pregnancy and lactation increased IL-6 expression, decreased expression of IL-1b, and had no effect on TNF-α mRNA expression in the nucleus accumbens (NAc) shell of the brain in male offspring^46^. However, another study have shown that feeding with a CAF diet 8 weeks before pre-pregnancy and during pregnancy increased IL-6 expression in placenta but did not alter inflammatory markers in the liver of fetuses on day 21 of pregnancy^45^.

Together, studies showed that female CAF diet consumed before and during pregnancy and/or lactation influences metabolic and inflammatory profiles and body composition of their offspring. However, there are only few reports on sex-specific differences in effects of diet in offspring and data are contradictory^35–37,47^.

We hypothesized that rats fed CAF diet before and during pregnancy and during lactation differently influence metabolic profiles, fat content and inflammation parameters in male and female offspring.

## Results

### Developmental data

#### CAF diet in mothers increased food intake during pregnancy but did not altered body weight and fat content before, during and after pregnancy

**Figure 1:**
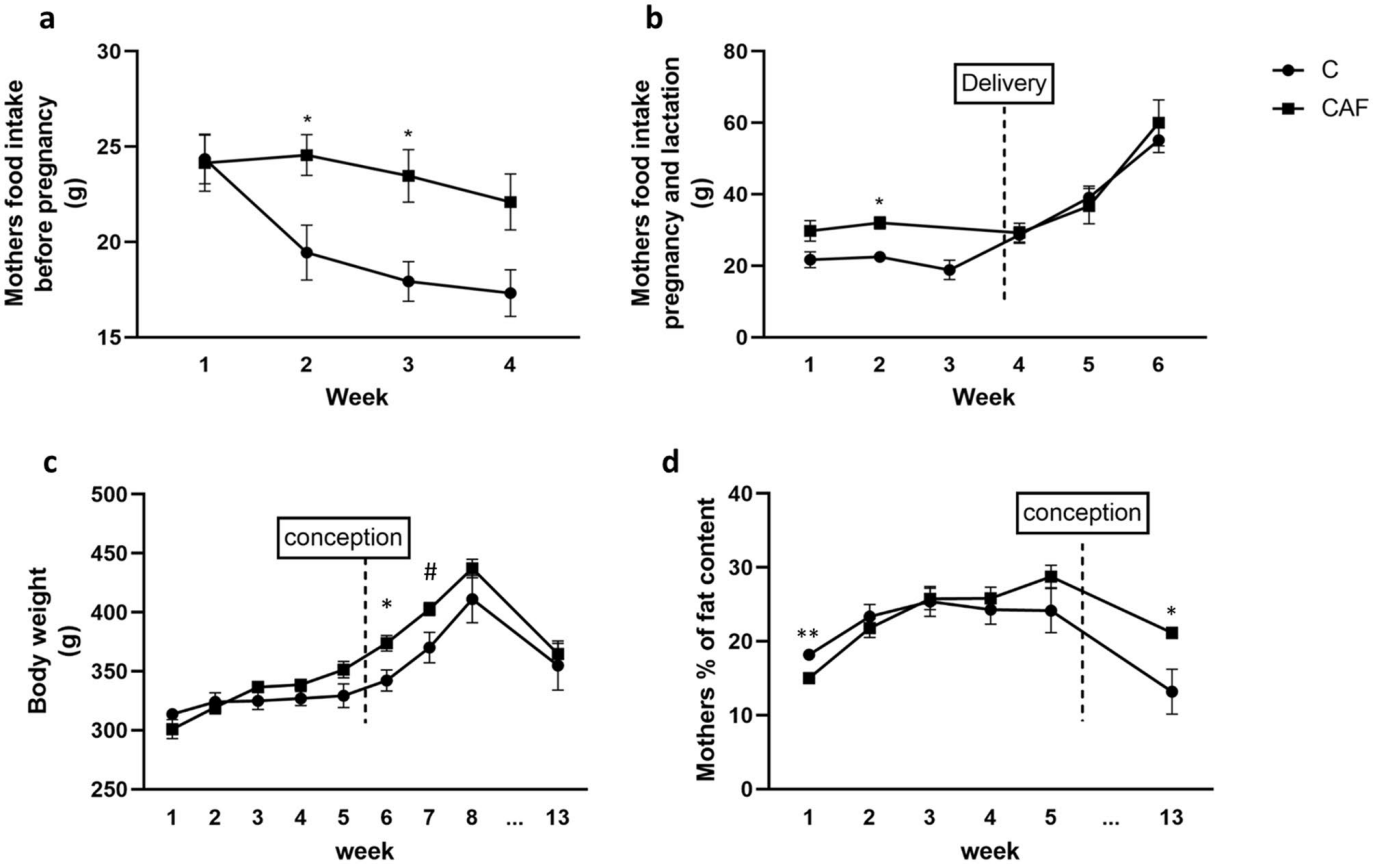
Mothers food intake before pregnancy and during pregnancy and lactation, and maternal body weight and fat content. (**a**) Weekly average of food intake in CAF and C groups before pregnancy; at 2 and 3 weeks CAF mothers had a significantly higher food intake (p’s < 0.05); (**b**) weekly average of food intake during pregnancy and lactation. At the 2nd week CAF mothers had significantly higher food intake (p < 0.05); (**c**) mothers weight in weekly time points: before pregnancy, during pregnancy, and at the end of the experiment; (**d**) percentage of fat content in mothers measured weekly before pregnancy and at the end of the experiment; black square—CAF females, black dot—C females (*p < 0.05; **p < 0.01; ^#^p = 0.06); n = 3 mothers/group*.*

There were significant differences in food intake in mothers in second and third weeks of administration of a CAF diet before pregnancy (p’s < 0.05; Fig. 1a). We have found that during these weeks mothers from the CAF group ate more compared to the C group. Moreover, there was difference in food intake in the second week of pregnancy, when CAF mothers ate more compared to C females (p < 0.05; Fig. 1b). We have also reported that after 6 weeks of a CAF diet females were heavier compared to controls (p < 0.05) The same trend was seen in week seven (p = 0.06) (Fig. 1c).

In terms of fat content, at the be beginning of the experiment—first week C females had higher fat content than CAF, but at the week 13th CAF females had higher fat contents compared to C animals (p < 0.05; Fig. 1d).

#### Effect of CAF diet on fat volume in mothers before and after pregnancy and lactation

**Figure 2:**
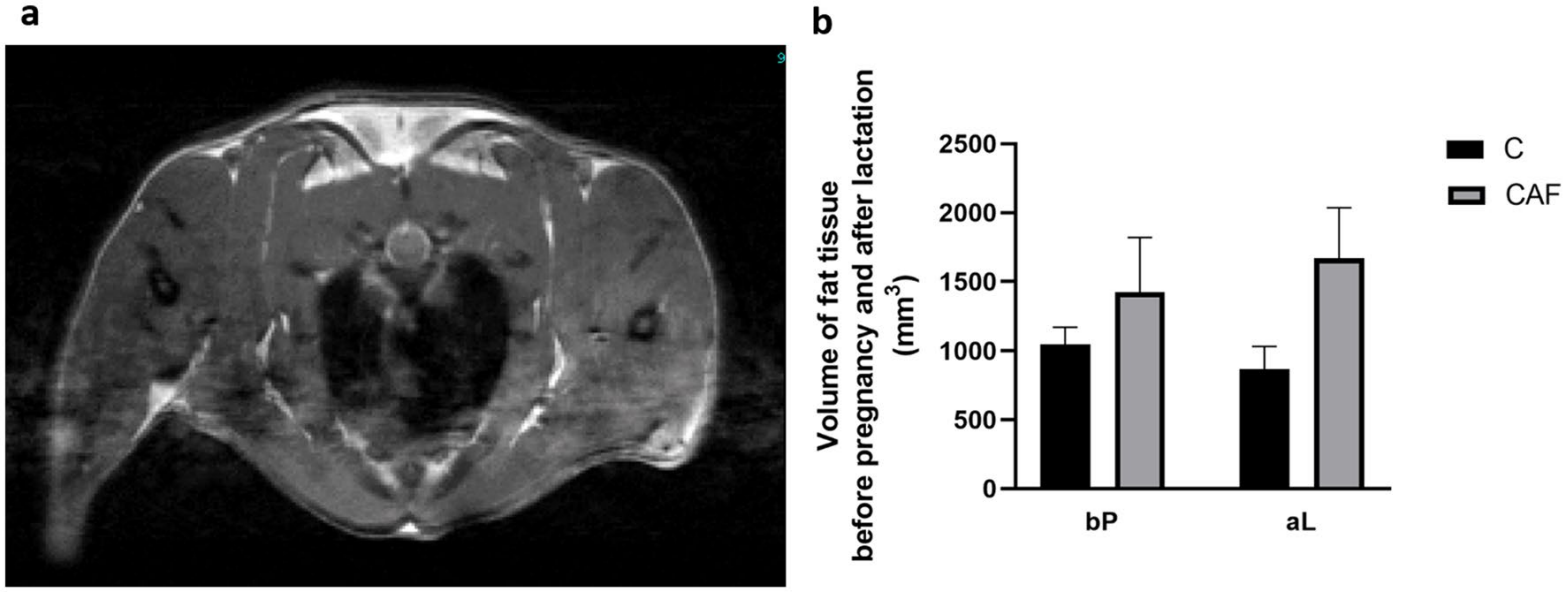
Magnetic resonance image. (**a**) A representative magnetic resonance image from C group; white arrow points an area of measurement of fat tissue; (**b**) volume of fat tissue measured before pregnancy and after lactation; *bP* before pregnancy (p = 0.42), *aL* after lactation (p = 0.12).

Figure 2a represents a sample magnetic resonance image obtained from measurement of fat tissue in C group . The volume of fat was measured by MRI before pregnancy and after lactation in CAF and C groups, and compared. There were no differences in volume of fat tissue in mothers before pregnancy (p = 0.42) and after lactation (p = 0.12) between CAF and C groups (Fig. 2b).

#### CAF diet did not influence body weight of pups on PND 3, but led to sex-specific alterations in body weigh in offspring and fat content on PND 25

**Figure 3:**
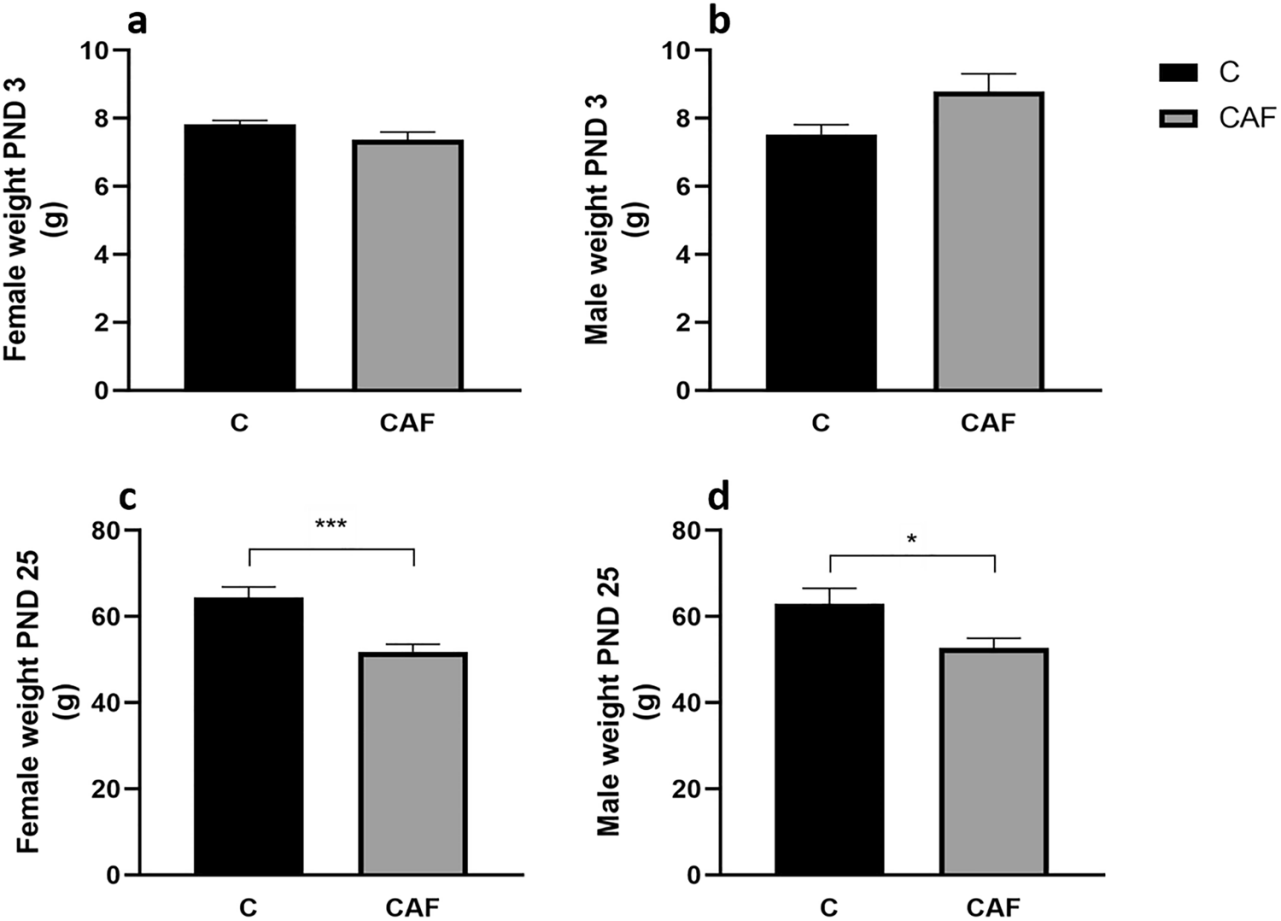
Weight of offspring at PND 3 and PND 25. (**a**) Comparison of weight of female offspring at PND 3 between CAF and C groups (p = 0.11); (**b**) comparison of weight of male offspring on PND 3 (p = 0.11); (**c**) comparison of weight of female offspring on PND 25 (***p < 0.001; females n = 9–13/group); (**d**) comparison of weight of male offspring on PND 25 (*p < 0.05; males n = 7–10/group); *C* control group, *CAF* cafeteria group.

There were no differences in offspring weight on PND 3 between groups (CAF and C) in both sexes (females: for CAF: 7.4 g ± 0.3, for C: 7.8 g ± 0.3; males: for CAF: 8.8 g ± 0.8, for C: 7.5 g ± 0.8; p’s = 0.11; Fig. 3a,b, respectively). However, on PND 25 both male and female offspring from mothers kept on CAF diet had lower weight compared to control. Additionally, we found marked sex differences in body weight of pups. Females offspring from CAF group weighed about 20% less compared to controls (for CAF: 51.8 g ± 2.9; for C: 64.4 g ± 2.9; p’s < 0.001; n = 9–13; Fig. 3c). Whereas, male offspring from CAF group weighed about 16% less than these from C group (for CAF: 52.7 g ± 4.0; for C: 63.0 g ± 4.0; p’s < 0.05; n = 7–10; Fig. 3d).

**Figure 4:**
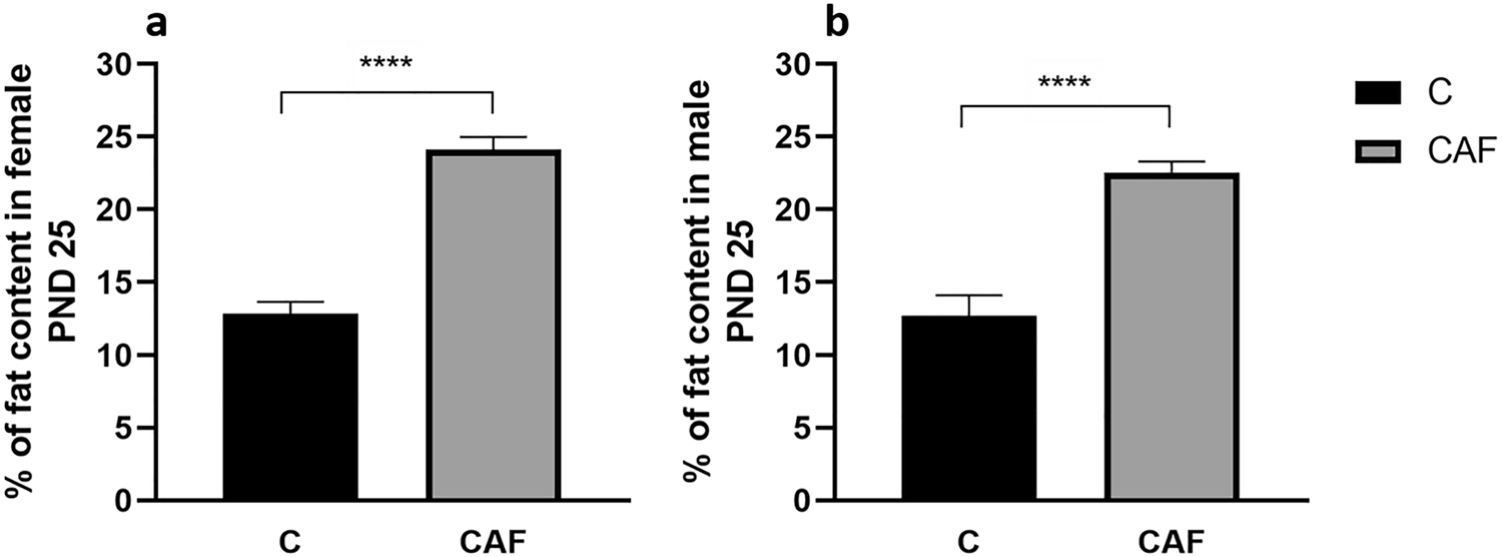
Percentage of fat content in offspring on PND 25. (**a**) Percentage of fat content in female offspring on PND 25 (****p < 0.001; females n = 9–13/group); (**b**) percentage of fat content in male offspring on PND 25 (****p < 0.001; males n = 7–10/group); *C* control group, *CAF* cafeteria group.

There were also significant differences in percentage of fat in offspring on PND 25. Both female and male offspring from mothers kept on a CAF diet had higher percentage of fat compared with controls (females: for CAF: 24.1 g ± 1.3, for C: 12.8 g ± 1.3, n = 9–13; males: for CAF: 22.5 g ± 1.5, for C: 12.7 g ± 1.5, n = 7–10; p’s < 0.001; Fig. 4a,b, respectively).

#### CAF diet altered metabolic parameters in offspring on PND 25 in a sex-specific manner

**Figure 5:**
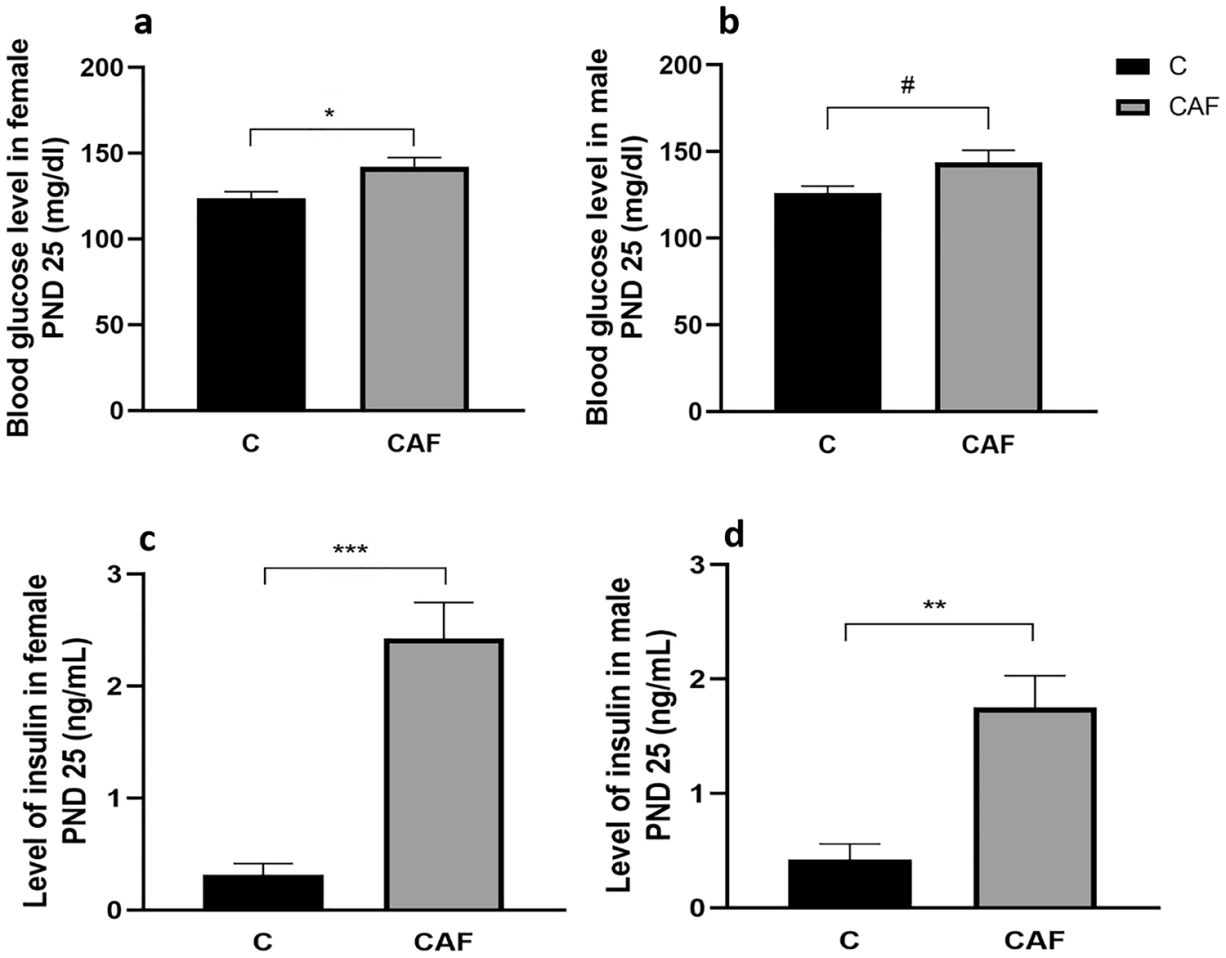
Metabolic status in offspring on PND 25. (**a**) Blood glucose level in female offspring (*p < 0.05); (**b**) blood glucose level in male offspring (^#^p = 0.07); (**c**) blood serum level of insulin in female offspring (***p < 0.005); (**d**) blood serum level of insulin in male offspring (**p < 0.01); *C* control group, *CAF* cafeteria group; n = 5/sex/group.

Female, but not male offspring from mothers kept on a CAF diet had a 14.5% higher blood glucose level when comparing to respective C groups (females: for CAF: 141.8 mg/dl ± 7.5, for C: 123.8 mg/dl ± 7.5; p < 0.05; Fig. 5a; males: for CAF: 143.8 mg/dl ± 9.1, for C: 126.0 mg/dl ± 9.1; p = 0.07; Fig. 5b; n = 5/sex/group).

In contrast to glucose data, both female and male offspring from CAF mothers had a robust increase in levels of insulin in blood serum compared to respective C groups. Additionally, we have reported that these differences were more pronounced in females. There was an 8 time increase in this hormone levels in female CAF offspring, whereas in males there was a 4.5 time rise, compared to relative controls offspring (females: for CAF: 2.4 ng/ml ± 0.3, for C: 0.3 ng/ml ± 0.3; p < 0.005; Fig. 5c; males: for CAF: 1.8 ng/ml ± 0.3, for C: 0.4 ng/ml ± 0.3; p < 0.01; Fig. 5d; n = 5/sex/group).

#### CAF diet had sex-specific effects on inflammatory parameters in offspring on PND 25

**Figure 6:**
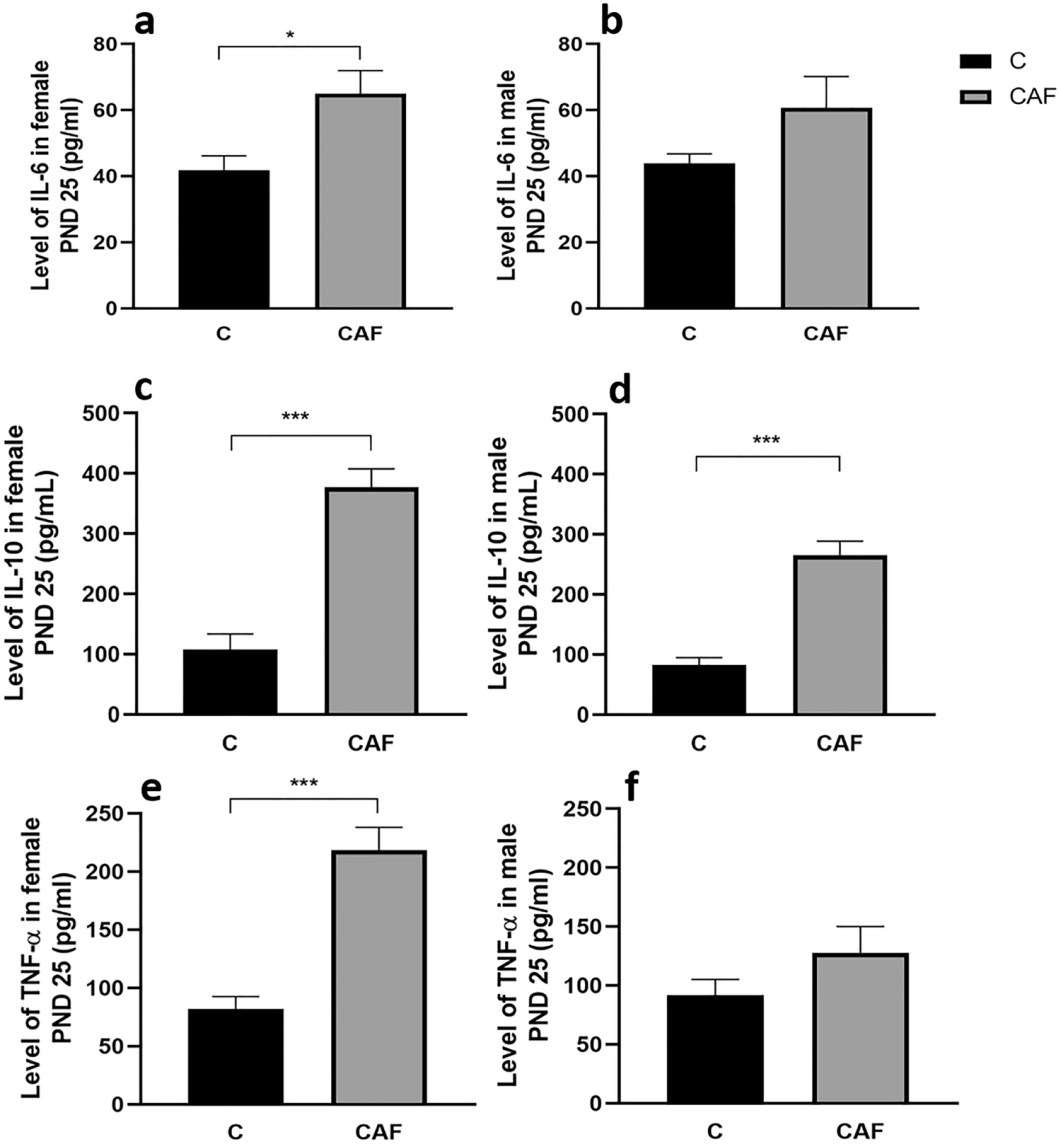
Inflammation status in offspring on PND 25. (**a**) Level of interleukin 6 (IL-6) in female offspring (*p < 0.05); (**b**) IL-6 level in male offspring (p = 0.12); (**c**) level of interleukin 10 (IL-10) in female offspring (***p < 0.005); (**d**) blood serum level of IL-10 in male offspring (p < 0.005); (**e**) level of tumor necrosis factor alpha (TNF-α) in female offspring (***p < 0.005); (**f**) level of TNF-α in male offspring (p = 0.20). *C* control group, *CAF* cafeteria group; n = 5/sex/group for all measurements.

We have also observed sex-specific effects of mothers’ CAF diet on inflammatory parameters in offspring. There were differences in IL-6 level in blood serum in offspring from mothers kept on a CAF diet, when compared to C group. However, these differences reached statistically significant values in female (a 14% increase), but not in male offspring (females: for CAF: 65.0 pg/ml ± 8.3, for C: 41.8 pg/ml ± 8.3; p < 0.05; Fig. 6a; males: for CAF: 60.7 pg/ml ± 9.8, for C: 43.9 pg/ml ± 9.8; p = 0.12; Figs. 6b; n = 5/sex/group).

In contrast to IL-6, both female and male offspring from mothers kept on CAF diet had significantly higher concentration of IL-10 in blood serum compared to respective C groups. However, again we found that this increase was higher in females compared to males (females: a 3.5 time increase; for CAF: 376.8 pg/ml ± 40.4, for C: 107.8 pg/ml ± 40.4; p < 0.005; Fig. 6c; males: a 3.2 time rise; for CAF: 265.6 pg/ml ± 28.3, C: 82.5 pg/ml ± 28.3; p < 0.005; Fig. 6d; n = 5/sex/group).

Finally, similarly to IL-6 data, levels of TNF-α in blood serum were significantly higher (2.7 times) in females but not males offspring from mothers kept on CAF diet when compared to C groups (females: for CAF: 218.5 pg/ml ± 23.9, for C: 82.3 pg/ml ± 23.9; p < 0.005; Fig. 6e; males: for CAF: 127.8 pg/ml ± 26.1, for C: 91.5 pg/ml ± 26.1; p = 0.20; Fig. 6f; n = 5/sex/group).

## Discussion

We confirmed the hypothesis that mothers' cafeteria diet differently influences metabolic profile, fat content and inflammation parameters in male and female rat offspring. Besides studying pups, we have also examined changes in females before pregnancy, during pregnancy, and during lactation. We have found that dams on a CAF diet had increased food intake in the second and the third week before pregnancy and in the second week of pregnancy when compared to C group. These findings are in agreement with data obtained by Sanchez‑Blanco et al., which have shown that female rats kept on a CAF diet 22 days before pregnancy and during pregnancy had higher food intake than control^48^.

In our study, in spite of a currently reported increase in food intake in dams, no statistically significant differences in mothers’ weight, and fat content were noticed. However, inspection of the figure (Fig.  1c) indicates a non-significant increase in body weight in dams in most of the time points, except of week 6th of a CAF diet administration. These non-significant effects may be a result of small groups in our study (n = 3 mothers/group). Indeed, studies on a bigger number of dams (n = 16/group) have shown that 6–13 weeks of exposure of rats to a CAF diet induced an increase in body weight in these animals^36^. Additionally, it was found that CAF rats gained extra 22% in weight during the pre-pregnancy period compared to controls, and were heavier during pregnancy. Moreover, CAF diet-fed dams still remained heavier than animals fed a chow diet at mid-lactation^36^.

Our studies also confirmed that both MRI and NMR are useful, non-invasive techniques in the assessment of fat contents and body composition, which could be successfully employed in studies of effects of a CAF diet on development of obesity. However, one limitation of MRI studies in anesthetized animals is heart and breathing movements, which unable to examine abdominal fat. Thus, in this study we could only examine volume of fat tissue on the neck of animals. Here, due to logistic considerations, we were not able to perform MRI studies in pups. However, in the future, we plan perform experiments using MRI on pups to examine changes in fat content at different time points of postnatal development.

The main aim of this study was, however, to examine influence of the CAF diet on pups and study possible sex differences in response to this metabolic insult.

We have found that on PND 3 there was no difference in body weight between pups on CAF and C diets. Such differences were revealed by PND 25 when both males and females offspring from mothers kept on CAF diet had lower weight compared to control. Additionally, we have found that at PND 25 both pups of both sexes from mothers kept on a CAF diet had higher fat contents. Thus, in our study we have confirmed reported by Pomar et al.^37^ the thin-outside-fat-inside phonotype in pups. Moreover, we have revealed that these differences were more pronounced in female offspring.

Cardenas-Perez et al. have shown that offspring from mothers kept on a CAF diet for 9 weeks (including pre-mating, pregnancy, and lactation) had slightly lower body weight at birth comparing to C group^49^. Moreover, when offspring was fed a CAF diet after weaning, this decrease in body weight persisted until 7th week of age. In the study  performed by Sanchez-Blanco et al.^48^ body weight at birth in pups from mothers kept on a CAF diet 22 days before pregnancy and during pregnancy was also lower, compared to control. Additionally, when pups remained on a CAF diet, they continued to be lighter until PND 21^48^. Similarly, in experiments conducted by Bayol^35^, it was revealed that when a CAF diet was given during gestation and lactation to mothers, it caused a decrease in body weight of offspring both at birth and at weaning (PND 21). However, when such a diet was continued up to week 10 postnatally, an increase in body weight both in males and females was reported^35^. Thus, our data and studies discussed above indicate that a CAF diet given to mothers before pregnancy, during pregnancy and lactation leads to a decrease in body weight in offspring. Lower body weight of offspring exposed prenatally to a CAF diet may be related to decreased protein intake by approximately 37% and 34% during gestation and lactation, respectively^50^.

Our study has also shown that in CAF female offspring at PND 25, a decrease in body weight is more pronounced compared to CAF male offspring. Sex differences in body weight of offspring was also reported in the experiment during which a mixture of a high-fat (HFD) and a CAF diet (HFD + CAF) was given to rats before mating, and during pregnancy^51^. Male but not female offspring from HFD + CAF groups had lower weight on PND 1. However, on PND 19, both male and female offspring form mothers on HFD + CAF diet, which remained on such a diet after delivery, had higher body weight compared to C group^51^.

Numerous experiments including our current study also confirm that exposure to a CAF diet: (1) before pregnancy and during pregnancy and lactation, (2) during pregnancy and lactation, (3) only during lactation leads to accumulation of fat tissue in offspring^37,38,52,53^.

Sex-specific differences were also found in blood glucose and insulin levels. On PND 25 only in females from CAF mothers there was a significant increase in blood glucose level. However, in case of insulin, concentration of this hormone was significantly higher both in male and female offspring form mothers kept on a CAF diet. However, again, this rise in insulin level was more pronounced in female offspring.

George et al. have shown that both sexes of Wistar rats’ offspring from mothers kept on a CAF diet during pregnancy, had no changes in fasting serum glucose levels at the age of 12 weeks. However, when dams were exposed to a CAF during lactation, the offspring had significantly higher level of fasting serum glucose compared to control^36^. Moreover, only male offspring from mothers kept on a CAF diet during lactation had higher peak of glucose during the glucose tolerance test at 12 weeks^36^. Pomar et al. have also shown that offspring at 3 and 6 weeks of age from mothers kept on CAF diet during lactation had impaired response to glucose tolerance test^37^. Furthermore, study of George^36^ and Pomar^37^ found no difference in plasma insulin levels between CAF and C offspring. However, George et al.^36^ have shown that, overall, male offspring had significantly higher level of plasma insulin compared with females, particularly at baseline and 2 h. Thus, the Authors confirm previous findings that exposure to a CAF diet during lactation caused male offspring to be more susceptible to insulin resistance^54,55^. It was also concluded that only postnatal exposure to maternal obesity lead to adiposity and insulin resistance. It was proposed that the milk composition of the dams is a key factor for programming effects. In our study, offspring remained on a CAF diet while in utero as well as during lactation, which prevents us to dissociate between prenatal and postnatal effects of the diet. Additionally, we have studied offspring in a much younger age (PND 25). However, similarly to above-mentioned studies, we have also revealed sex-specific effects of a CAF diet on metabolic outcomes.

Effects of a CAF diet on glucose levels were also studied in offspring. Bayol et al. revealed that exposure of Wistar rats to a CAF diet during pregnancy, lactation, and post-weaning period leads to sex differences in both glucose and insulin levels in offspring^35^. Similarly to our results at PND 25, females at the age of 10 weeks from mothers on a CAF diet had higher concentration of glucose level when compared to control^35^. However, both in Bayol’s^35^ and our studies, there was no difference in glucose levels between male offspring form CAF and C mothers. On the other hand, concentration of insulin was significantly higher in male offspring in comparison to control, but with no difference in female offspring at the age of 10 weeks^35^. In our study, with a shorter exposure to a CAF diet, raised level of this hormone was seen both in male and female offspring. Thus, additional feeding during post-weaning period with a CAF diet may contribute to increased levels of insulin observed in males in the age of 10 weeks. However, study performed by Bayol^35^ also indicates that CAF-induced adiposity and metabolic disruptions were increased in adult offspring from mothers exposed to such a diet during pregnancy and lactation, when compared with offspring only fed the diet after weaning. Indeed, it was shown that adipocyte hypertrophy and increase in perirenal fat pad mass relative to body weight persist, even when the offspring were fed a chow diet after weaning.

Here we have provided novel findings on sex-specific differences in inflammatory parameters in offspring in response to a maternal CAF diet. Significant increase of interleukin 6 (IL-6) and tumor necrosis factor alpha (TNF-α) was found in females from CAF mothers, but not in male offspring. In contrast, significant elevation of interleukin 10 (IL-10) was found in both sexes of offspring. However, concentration of IL-10 in females from CAF mothers was higher than in male offspring. Our results are in agreement with studies performed on adult rats exposed to a CAF diet. CAF diet leads to TNF-α overexpression in the intestine^56^ and in plasma^33^ of adult male rats^5^. CAF-fed adult rats also displayed remarkable inflammation in white fat, brown fat and liver^33^. Both in animal and human obesity, low-level chronic inflammation and macrophage infiltration into adipose tissue is a well-documented phenomenon^57–59^. However, signs of inflammation were found not only at the periphery but also centrally, as consumption of a CAF diet increases both peripheral and central levels of interleukin-1β (IL-1β), a pro-inflammatory cytokine. Eighteen weeks of administration of a Western diet elevated blood serum level of TNF-α and decreased levels of IL-10 and IL-6 in in male Wistar rats, when compared to control^44^. However, shorter, 6-week administration of a CAF diet to male adult rats had no significant influence of diet on inflammatory parameters (TNF-α and IL-6)^34^. However, longer administration of a CAF diet (15 weeks) to adult mice lead to inflammatory processes, characterized by significantly higher concentration of IL-6^25^. Thus, duration of the administration of a CAF diet may be a key parameter when discussing inflammatory responses. In contrast to well-documented inflammation caused by a CAF diet in adult animals, literature on this subject in prenatal models is spare. When a CAF diet was given to rat mothers 8 weeks before pregnancy and during pregnancy, it did not increase the inflammatory status of the mother, placenta or fetus in late gestation (PND 21)^45^. Actually, levels of inflammatory markers such as IL-6, IL-12p40 and MIP2 were reduced slightly. Carillon et al. have shown that Wistar rat offspring from mothers kept on CAF diet for 9 weeks, including pregnancy and lactation, had higher mRNA expression of IL-6 in brain than C. However, differences in TNF-α mRNA expression were not observed^23^.

In spite of the fact that CAF diet is commonly used in many laboratories, the main caveat refers to its nutritional composition, which is uncontrolled, given that the animals can choose among a variety of different foods^60^. There is no standard CAF diet protocol, its caloric content and selection of products varies largely between studies^61^. Additionally, percentage of energy derived from fat and carbohydrates differs and ranges between 17–60 and 37 and 73, respectively. Moreover, while in some studies CAF was used alone, in other it has been combined with a standard chow diet. However, one way to overcome these difficulties is to simplify the diet, and employ it with only 3 or 4 food choices^62,63^, which were used in the current study. Finally, protocols used by different researches varied in time of exposure to the diet.

In summary, we have shown that mothers exposure to a CAF diet, which mimics a “Western-style diet” before and during pregnancy and lactation increased body weight, fat contents, changed metabolic profiles (levels of glucose and insulin), and led to inflammation in offspring. Moreover, we have revealed that above alterations are sex-specific and more pronounced in female offspring. We have also shown that non-invasive imaging techniques such as NMR and MRI could be successfully employed in a CAF-induced obesity model. Our study indicates a crucial need to monitor early signs of development of metabolic problems in offspring form mothers kept on a CAF diet with respect to sex differences.

## Materials and methods

### Breeding of animals

**Figure 7:**
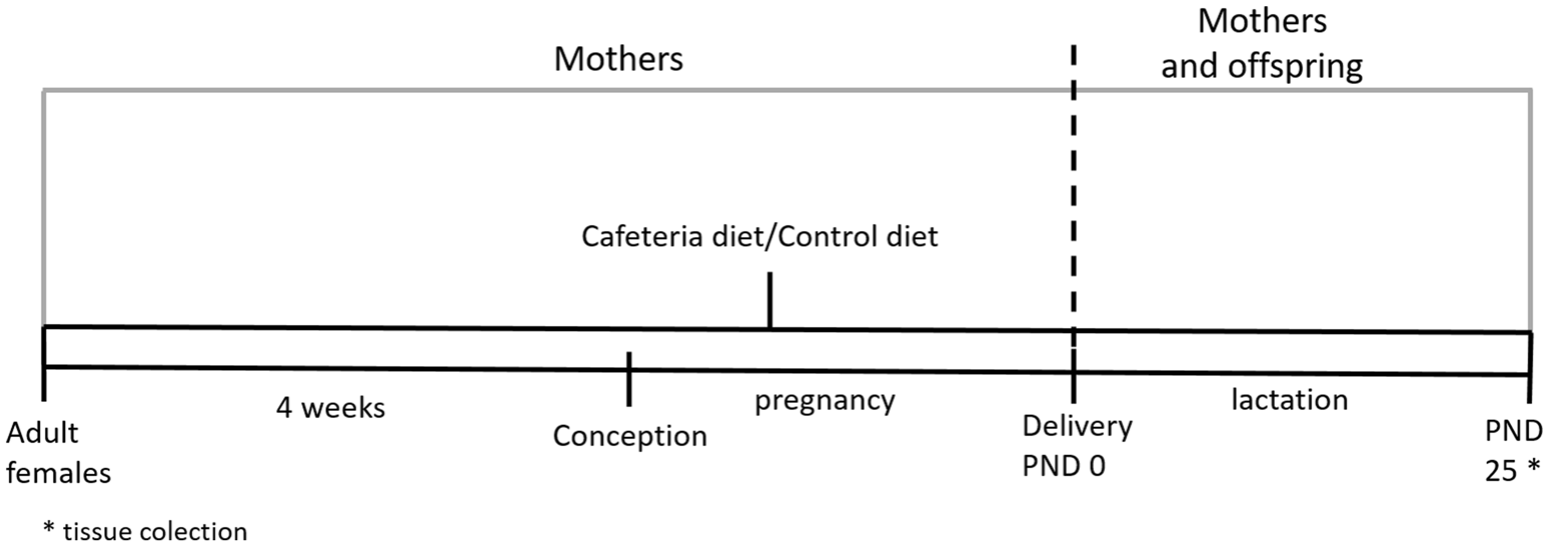
Experimental design. After 4 weeks of feeding with cafeteria (CAF) or control (C) diets, adult female rats were fertilized (n = 3). Administration of CAF diet was contained during pregnancy and lactation until postnatal day 25 (PND 25).

Six 2-month-old female Wistar rats (300 g ± 13 g) and three 2-month-old male Wistar rats were obtained from the licensed Animal Breeding Company in Poznan. Rats were housed in cages under constant conditions of light/dark cycle 12/12 h and temperature (21 °C). Animals had ad libitum access to water and a standard laboratory chow diet (AIN93G, Zoolab, Poland). After 1 week of acclimatization, rats were divided into two groups: (1) cafeteria diet (CAF; n = 3; animals fed with composed diet; see Table 1 for used products and their nutritional values) and control (C; n = 3; animals fed a standard chow diet AIN93G, Zoolab, Polska). Females from CAF group were fed a CAF diet for 4 weeks before pregnancy, as well as during pregnancy and lactation. Similarly, a standard chow diet was administrated to C group for the same amount of time. Both groups had ad libitum access to water and food during experiment (Fig. 7). Experiment was approved by the Local Ethics Committee for the Experiments on Animals, Poznan University of Life Sciences, Poland (license no. 55/2018; 38/2020). All experiments were performed in accordance with relevant guidelines and regulations. Reporting in the manuscript follows the recommendations in the ARRIVE guidelines. After 4 weeks of administration of CAF or C diet, six adult females were paired with three adult males for fertilization. Males and females were kept in wire-bottom cages for 2 days. Pregnancies were confirmed by the presence of vaginal plugs. After fertilization, females were kept in separate cages during the whole pregnancy and lactation with ad libitum access to food, water and nest material (Fig. 7).

**Table 1:** Products and their nutritional values of CAF diet composition.

Nutritional values per 100 g of product						
Products	Company	Energy (kcal)	Fat (g)	Cabrohydrates (g)	Protein (g)	Salt (g)
Cheese	Mlekpol	347.0	27.0	0.0	26.0	1.2
Dried sausages	Tarczyński	538	47.0	4.8	24.0	3.1
Biscuits	Krakuski	440.0	13.0	71.0	8.9	1.1
Chocolate	E. Wedel	501.0	28.0	52.0	6.0	0.1
Peanut butter	Nusskati	563.0	37.0	49.0	6.5	0.1
Chips	Lays’	536.0	33.0	52.0	5.9	1.1
Cookies	E. Wedel	358.0	7.0	70.0	3.2	0.2
Pate	Duda	259.0	23.0	3.2	9.8	1.9
Jam	Łowicz	142.0	0.5	35.0	0.5	0.0
Choccolate bars	Nestle	488.0	21.6	68.5	4.1	0.5
Dried bananas	Bakalland	530.0	34.0	50.0	2.0	0.0
Muffins		431.0	24.0	47.0	6.1	0.6

### Composition of the cafeteria (CAF) diet

The CAF diet were composed by experimenters based on earlier published studies using that kind of a diet^3,24^. The CAF diet consists of 12 different products: cheese, dried sausage, biscuits, chocolate, peanut butter, potato chips, cookies, pate, jam, chocolate bar, dried bananas and muffin (Table 1). Products and their nutritional values in a CAF diet are presented in Table 1. Every day, each female from the CAF diet group received three products (two sweet and one savoury). Food portions were weighed on a laboratory scale, and products were changed daily and in threes, so that these three do not repeat. Rats had ad libitum access to a CAF diet and tap water during the whole experiment. Remaining food was weighed daily and food intake was calculated.

### Body composition test

Females from both groups (CAF and C) were analyzed weekly (except pregnancy and lactation period) by nuclear magnetic resonance (NMR) using Minispec LF90 (Billerica, Massachusetts, USA). It is a non-invasive and short time analysis, in which weight of an animal as well as a whole animal body composition (absolute weight of fat and free body liquid) are measured. The NMR technique does not require administration of anesthetics, which makes it safe and not stressful for animals. Data obtained from NMR were analyzed and percentage of fat content in each time point was calculated.

### MRI scanning

After 4 weeks of administration of diets dams from CAF and C groups were subjected to MRI scanning. MRI experiments were carried out using a preclinical horizontal scanner operating at 9.4 T (400 MHz—Agilent) equipped with a 600 mT/m gradient system. For the MRI imaging, a 72-mm i.d. quadrature birdcage type coil was used.

During the MRI experiment, animals were put at specially designed holder and anesthetized with 1.5–2% isoflurane in a 50/50 air-oxygen mixture. The temperature of the animal was kept at 37 °C. Respiration of the animal were monitored and used to synchronize MRI experiments.

MRI images of spin density were collected at the location of fat tissue using fast spin echo technique (FSEMS) with parameters: TR = 5 s, effective TE = 10 ms, ETL = 8, FOV 62 × 62 mm, matrix size 256 × 256, 18 slices. Next, at the same location FSEMs sequence with additional fat saturation was used.

Collected data with and without fat signal were transformed to DICOM format and images obtained from the second experiment were subtracted from collected images using ImageJ software. Then, the Volumest (ImageJ plugin) was employed to count volumes of fat for every animal in three independent trials.

### Offspring

Pups were born at 22–23 day of pregnancy. On postnatal day 3 (PND 3), pups were weighed on a laboratory scale, and their sex was identified. The sex check was guided by the distance between anus and urethra. On PND 3 litters were unified to 8 pups per litter.

### Sample collection

At PND 25, animals were analyzed by NMR to measure body composition (weight, absolute fat and free body liquid content). Then, animals were sacrificed using the CO_2_ chamber (Equipement Veterinaire Minerve, Esternay, France), decapitated, trunk blood was collected, and blood serum was frozen in liquid nitrogen, and stored at − 80 °C for further testing. Because of the small amount, blood serum of offspring was pooled to n = 5/sex/group.

### Metabolic profiles of offspring

Levels of glucose in pups were measured by AccuChek Active device (Roche Diabetes Care, Warsaw, Poland). Offspring’s insulin level in blood serum was measured by radioimmunoassay rat RIA kit (Rat insulin RIA test RI-13K, Merck).

### Inflammatory profiles of offspring

Levels of interleukins: IL-6 and IL-10, as well as tumor necrosis factor alpha (TNF-α) in blood serum were measured using immune-enzymatic ELISA kits (Elabscience, Wuhan, Hubei, China).

### Statistical analysis and data presentation

Mothers' body weight, fat content, and food intake were analyzed using analysis of variance (ANOVA) for the factors of group (CAF and C), with day as a repeated measure. Maternal adipose tissue volume, offspring weight, fat contents, and metabolic and inflammatory parameters were analyzed using unpaired t-test, compering CAF and C groups. For the measurements of fat volume from MRI scans, ImageJ software (https://imagej.nih.gov/ij/) and Volumest (ImageJ plugin) were used. Graphs were prepared using Graph Pad Prism 8 software (GraphPad Software, San Diego, USA). Data are presented as means ± SEM. P values of less than 0.05 were considered statistically significant. All statistical analyses were performed using Graph Pad Prism 8 software (GraphPad Software, San Diego, USA).

### Ethics approval

The animal experiments were approved by the Animal Ethics Committee at the Poznan University of Life Sciences.
